# Essential Oils and Eugenols Inhibit Biofilm Formation and the Virulence of *Escherichia coli* O157:H7

**DOI:** 10.1038/srep36377

**Published:** 2016-11-03

**Authors:** Yong-Guy Kim, Jin-Hyung Lee, Giyeon Gwon, Soon-Il Kim, Jae Gyu Park, Jintae Lee

**Affiliations:** 1 School of Chemical Engineering, Yeungnam University, Gyeongsan 38541, Republic of Korea; 2 Nareso Research Center, Seoho-ro 89, Suwon 16614, Republic of Korea; 3 Pohang Center for Evaluation of Biomaterials, Pohang Technopark Foundation, Pohang 37668, Republic of Korea

## Abstract

Enterohemorrhagic *Escherichia coli* O157:H7 (EHEC) has caused foodborne outbreaks worldwide and the bacterium forms antimicrobial-tolerant biofilms. We investigated the abilities of various plant essential oils and their components to inhibit biofilm formation by EHEC. Bay, clove, pimento berry oils and their major common constituent eugenol at 0.005% (v/v) were found to markedly inhibit EHEC biofilm formation without affecting planktonic cell growth. In addition, three other eugenol derivatives isoeugenol, 2-methoxy-4-propylphenol, and 4-ethylguaiacol had antibiofilm activity, indicating that the C-1 hydroxyl unit, the C-2 methoxy unit, and C-4 alkyl or alkane chain on the benzene ring of eugenol play important roles in antibiofilm activity. Interestingly, these essential oils and eugenol did not inhibit biofilm formation by three laboratory *E. coli* K-12 strains that reduced curli fimbriae production. Transcriptional analysis showed that eugenol down-regulated 17 of 28 genes analysed, including curli genes (*csgABDFG*), type I fimbriae genes (*fimCDH*) and *ler*-controlled toxin genes (*espD*, *escJ*, *escR*, and *tir*), which are required for biofilm formation and the attachment and effacement phenotype. In addition, biocompatible poly(lactic-co-glycolic acid) coatings containing clove oil or eugenol exhibited efficient biofilm inhibition on solid surfaces. In a *Caenorhabditis elegans* nematode model, clove oil and eugenol attenuated the virulence of EHEC.

Enterohemorrhagic *Escherichia coli* O157:H7 (EHEC) is responsible for outbreaks of hemorrhagic colitis and associated bloody diarrhea^1^. EHEC forms attaching and effacing (AE) lesions on human epithelial cells and produces Shiga-like toxins, which are responsible for the development of hemolytic-uremic syndrome^2^. Unfortunately, no effective therapy is available because antimicrobial agents increase the risk of developing hemolytic-uremic syndrome, a major cause of acute renal failure in children^1^.

The first stage of EHEC infection involves the adhesion of bacterial cells to host cells and the formation of microcolonies leading to colonization of the large intestine^2^. EHEC is also able to form biofilms on various biotic and abiotic surfaces, such as, on plants, stainless steel, glass, and polymers^3^,^4^. These biofilms are resistant to conventional antimicrobial agents, host defenses, and external stresses. Accordingly, in clinical and industrial environments EHEC biofilms pose a substantial challenge, and methods of controlling these biofilms are urgently required. The mechanism of EHEC biofilm formation is complex, which has been the subject of research. The importance of fimbriae, including curli and pili, for EHEC biofilm formation has been well-documented^4^,^5^,^6^. Swimming and swarming motilities influence the biofilm development of *E. coli*^7^,^8^,^9^. Also, several signaling molecules, such as cyclic di-GMP, autoinducer-2, and indole, are known to be involved in the biofilm formation of *E. coli*^7^,^10^.

Essential oils derived from plants have been widely used as antimicrobial and flavoring agents due to their variable terpenoid and phenolic contents^11^, and as a result, the anti-bacterial and anti-fungal activities of several essential oils have been well-studied^11^,^12^,^13^. However, traditional antimicrobial agents primarily designed to inhibit cell growth often result in bacterial drug resistance, and thus, essential oils have been studied using other developmental approaches, such as, the inhibition of biofilm formation, toxin production, bacterial quorum sensing, and of adhesive factors. For example, carvacrol and eugenol^14^, grapefruit limonoids^15^, *β*-sitosterol glucoside from clementine peel^16^, ginkgolic acids from *Ginkgo biloba*^17^, and cinnamaldehyde and eugenol from cinnamon bark oil^18^, which are found in essential oils, have been reported to inhibit EHEC biofilm formation. However, few studies have been undertaken to compare the antibiofilm characteristics of large numbers of essential oils.

In this study, 83 essential oils were initially screened for their ability to inhibit EHEC biofilm formation. Three oils, namely, bay, clove, and pimento berry oils demonstrated strong anti-biofilm activity against EHEC at sub-inhibitory concentrations, but not against laboratory *E. coli* strains. Chemical structure-activity assays revealed that eugenol and three other eugenol derivatives had anti-biofilm activity. In order to understand their action mechanisms, transcriptional analysis, motility analysis, and electron microscopy were utilized. In addition, a biocompatible poly(lactic-co-glycolic acid) surface coatings containing biofilm inhibitors were prepared and their antibiofilm effects were examined. Finally, an *in vivo Caenorhabditis elegans* model was used to study the effects of eugenol and of clove oil to confirm their antivirulence effects on EHEC.

## Results

### Anti-biofilm effects of essential oils against EHEC

**Figure 1:**
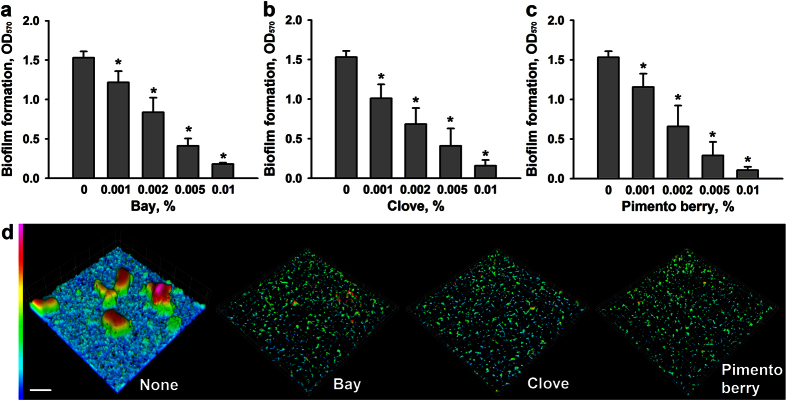
Effects of bay, clove, and pimento berry oils on EHEC biofilm formation. Biofilm formation (OD_570_) by EHEC was quantified in the presence of each of the three essential oils selected for further study after culture for 24 h in 96-well plates (**a**–**c**). Biofilm formation by EHEC/pCM18 tagged with green fluorescent protein in the presence of essential oils (0.005%) was confirmed by confocal laser microscopy (**d**). Scale bar = 50 μm.

To identify new anti-biofilm agents, 83 essential oils were initially screened in 96-well plates at a concentration of 0.005% (v/v) to minimize antimicrobial effects. Several essential oils were found to inhibit EHEC biofilm formation, but with widely different efficiencies. Detailed information on EHEC growth and biofilm formation in the presence of the 83 essential oils is provided in Supplementary Table S1. Notably, four essential oils, namely bay, cinnamon bark, clove, and pimento berry oil inhibited EHEC biofilm formation by more than 75%. No growth reduction of EHEC cells above 30% at OD_620_ was observed at 0.005% (v/v) as compared with untreated controls. Kim *et al.* found that cinnamon bark oil^18^ had antibiofilm activity against EHEC, but this is the first time that bay, clove, and pimento berry oils have been reported to have antibiofilm activity. In the present study, more detailed study showed bay, clove, and pimento berry oil all dose-dependently inhibited EHEC biofilm formation in 96-well polystyrene plates (Fig. 1a–c). Since bacteria form biofilms on the bottoms and sides of these plates, confocal laser microscopy and EHEC expressing green fluorescent protein were used to observe biofilm formation on glass, and our microscopic observations confirmed that all three essential oils dramatically inhibited biofilm formation on the bottom of glass (Fig. 1d). Biofilm inhibition was further confirmed by COMSTAT analysis. More specifically, bay, clove, and pimento berry oils reduced all three measured parameters (biomass, mean thickness, and substratum coverage) of EHEC (Table 1), and biomass (volume/area) and mean thickness were reduced by >80% by all three oils at 0.005% (v/v).

### Identification of the active anti-biofilm components in essential oils

**Figure 2:**
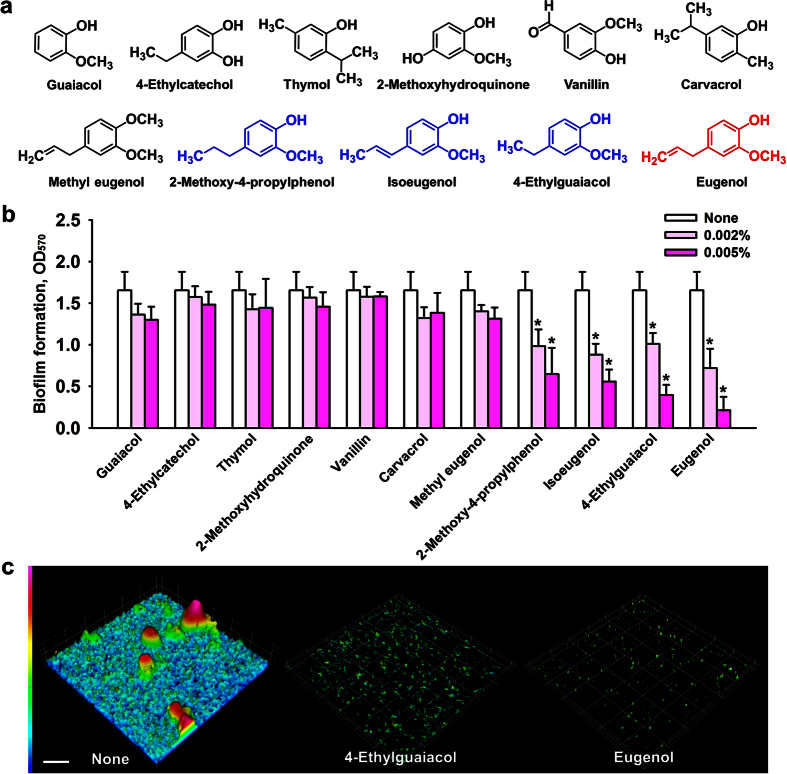
Inhibition of biofilm formation by eugenol-like compounds. Chemical structures are shown (**a**). Biofilm formation by EHEC was quantified in the presence of selected chemicals after incubation for 24 h in 96-well polystyrene plates without shaking (**b**). At least two independent experiments were conducted (total 12 wells). Error bars indicate standard deviations. Biofilms formed in the presence of 4-ethylguaiacol or eugenol (0.005%) were observed under a confocal laser microscope (**c**). Scale bar = 50 μm. *P < 0.05 versus non-treated controls.

To identify the active anti-biofilm components in the above three essential oils, GC-MS analysis was performed and as a result 33 different compounds were identified (Table 2). Eugenol was the predominant component and accounted for more than 62% of all three oils. In addition, myrcene, chavicol, methyleugenol, and *β*-caryophyllene were found to be present at >7.8%. This result concurs with previous reports, in which bay oil^19^, clove oil^20^, and pimento berry oil^21^ were found to contain about 60% eugenol. As was expected, eugenol was also found to dose-dependently inhibit EHEC biofilm formation (Fig. 2b), whereas other compounds, such as, myrcene and *β*-caryophyllene, did not show anti-biofilm activity at concentration under 0.005% (data not shown). These results suggest that eugenol is largely responsible for the anti-biofilm activity of these oils.

The antimicrobial activities of essential oils and eugenol were investigated by measuring minimum inhibitory concentrations (MICs) and EHEC planktonic cell growth. The MICs of the three oils and eugenol against EHEC were >0.1%, which is consistent with previously reported values^12^,^22^. Notably, MICs against EHEC were 20-times higher than the concentrations (~0.005%) required for antibiofilm activity. Furthermore, the three oils and eugenol at concentrations ≤0.005% did not retard planktonic cell growth, whereas at 0.01%, they reduced final cell density by about 20% (Supplementary Fig. S1). These findings indicate reduced biofilm formation by the three oils or eugenol was due to antibiofilm activity and not to antimicrobial activity.

### Anti-biofilm activities of eugenol derivatives on EHEC

To identify the structural motif present in eugenol responsible for antibiofilm activity against EHEC, we investigated the antibiofilm activities of eleven eugenol-related compounds (Fig. 2a). We found eugenol, 4-ethylguaiacol, isoeugenol, and 2-methoxy-4-propylphenol at 0.005% markedly inhibited EHEC biofilm formation by ≥50% versus untreated controls. Specifically, eugenol at 0.005% decreased EHEC biofilm formation by 87%, whereas the other seven compounds (guaiacol, 4-ethylcatechol, thymol, vanillin, carvacrol, methyl eugenol, and 2-methoxyhydroquinone) at concentrations of up to 0.005% did not significantly inhibit EHEC biofilm formation. Although, it has been previously reported that vanillin inhibits biofilm formation by *Aeromonas hydrophila*^23^, it did not inhibit EHEC biofilm formation in the present study.

Interestingly, antibiofilm activity against EHEC was found to be closely related to the presence and size of a C-4 alkane chain, and the presence of a hydroxyl group and a methoxy group at the C-1 and C-2 positions of the benzene ring, because eugenol, 4-ethylguaiacol, isoeugenol, and 2-methoxy-4-propylphenol all possess a C-4 alkane or alkyl chain and C-1 hydroxyl and C-2 methoxy groups (Fig. 2a). However, methyl eugenol, which has an alkyl chain and a methoxy unit at C-1 and C-2 lacked inhibitory activity, indicating the eugenol backbone and the C-1 hydroxyl and C-2 methoxy groups are required for antibiofilm activity. Because eugenol reduced biofilm formation most, we focused on it and eugenol-rich clove oil for further study.

Microscopic observations confirmed that eugenol and 4-ethylguaiacol at 0.005% (v/v) markedly inhibited EHEC biofilm formation (Fig. 2c). In addition, COMSTAT analysis confirmed that eugenol and 4-ethylguaiacol reduced biofilm biomass, mean thickness, and substratum coverage (Table 1).

### Effects of the three essential oils and eugenol on biofilm formation by other *E. coli* strains

**Figure 3:**
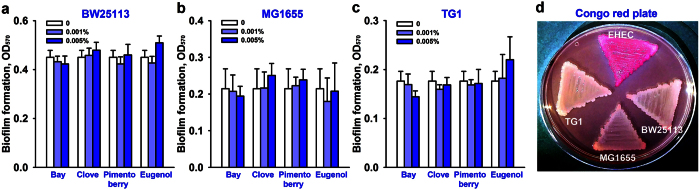
Effects of bay, clove, pimento berry oils and eugenol on biofilm formation by three laboratory *E. coli* strains and fimbriae production. Biofilm formation (OD_570_) by *E. coli* BW25113 (**a**), MG1655 (**b**), and TG1 (**c**) was quantified in the presence of essential oils or eugenol after culture for 24 h in 96-well plates without shaking. Fimbriae production was assessed on Congo red plates after 24 h (**d**). At least three independent experiments were conducted.

It is important that we develop therapeutic compounds that inhibit pathogenic biofilm formation but leave beneficial commensal biofilms unharmed^24^. Thus, the effects of the three essential oils and eugenol were investigated on three laboratory *E. coli* strains: BW25113, MG1655, and TG1. Unlike that observed for EHEC, neither eugenol nor the three oils had any biofilm inhibitory effects on these three *E. coli* K-12 strains (Fig. 3a–c). The three laboratory *E. coli* strains developed poor biofilms in comparison to the EHEC strain as judged by crystal violet staining. The biofilm formation of the untreated laboratory strains was similar to that of the EHEC strain treated with 0.005% eugenol. It is intriguing how eugenol specifically inhibits biofilm formation by EHEC. Because curli fimbriae are critical for *E. coli* biofilm development^25^, we investigated fimbriae productions by EHEC and the three laboratory strains. Interestingly, EHEC produced more fimbriae than the three laboratory *E. coli* strains on Congo red plates (Fig. 3d), which suggests eugenol inhibits the development of fimbriated *E. coli* like EHEC.

### Effect of the three essential oils and eugenol on EHEC motility

The impacts of three essential oils and eugenol on the swarming and swimming motilities of EHEC were also investigated because motility plays an important role in *E. coli* biofilm formation^9^,^26^. While the three oils clearly reduced swarming motility, eugenol did not (Supplementary Fig. S2a), and neither the three oils nor eugenol changed EHEC swimming motility (Supplementary Fig. S2b). Additionally, the presence of elongated swarming and swimming was measured as the diameter (in millimeters) of the zone of expansion (Supplementary Fig. S2c,d). The result suggests that the anti-biofilm effect of eugenol on EHEC is not closely related to motility.

### Transcriptional changes in EHEC cells induced by clove oil or eugenol

**Figure 4:**
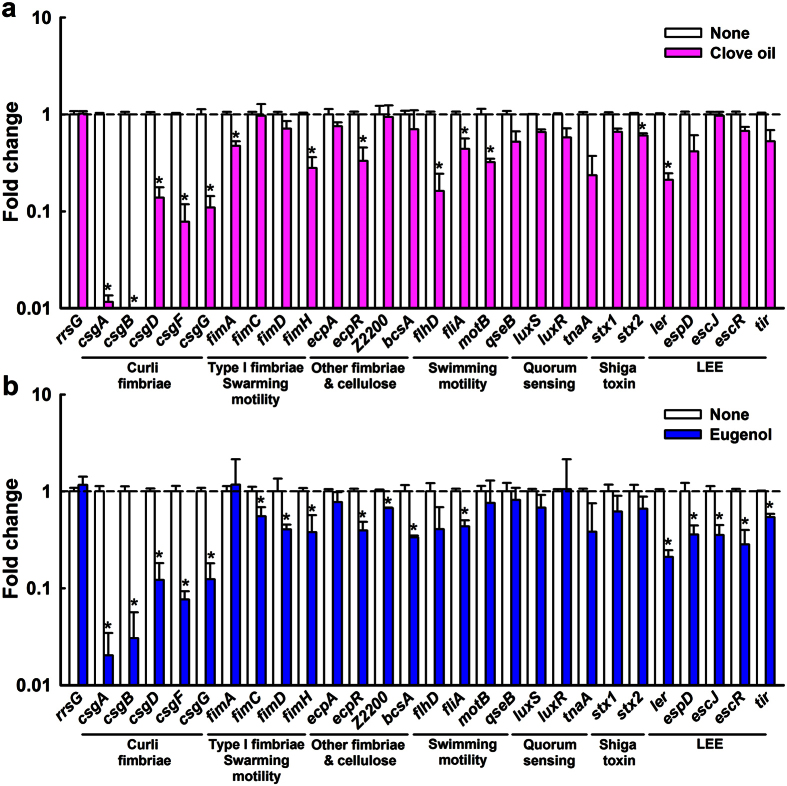
Transcriptional profiles of EHEC cells treated with or without clove oil or eugenol. EHEC was cultivated in LB broth with or without clove oil (**a**) or eugenol (**b**) at 0.005% (v/v) for 2 h with agitation (250 rpm) at 37 °C. Transcriptional profiles were measured by qRT-PCR. Relative gene expressions represent transcriptional levels after treatment with clove oil or eugenol versus untreated controls (value 1.0). The experiment was performed in duplicate.

To investigate the genetic bases of EHEC biofilm inhibition by clove oil and eugenol, qRT-PCR was performed to examine the differential expressions of biofilm- and virulence-related genes in treated and non-treated EHEC cells. Most noticeably, both clove oil and eugenol most markedly inhibited the expression of curli genes (*csgABDFG*) by 8-fold to 155-fold, while they less appreciably changed the expression of type I fimbrial and other fimbrial genes (Supplementary Table S2), whereas they did not affect the expression of *rrsG* (a housekeeping gene) (Fig. 4). Also, clove oil down-regulated several motility genes (swarming genes *fimA* and *fimH* and swimming genes *flhD*, *fliA*, and *motB*), and LEE transcriptional regulator *ler* gene (Fig. 4a), which concur with its inhibition of motility (Supplementary Fig. S2). On the other hand, eugenol altered the expression of motility genes less but decreased the expressions of *ler* and *ler*-controlled toxin genes (*espD*, *escJ*, *escR*, and *tir*) (Fig. 4b). Neither clove oil nor eugenol appreciably affected the expressions of quorum sensing genes (*luxS*, *luxR* and *tnaA*) or Shiga-like toxin genes (*stx1* and *stx2*). Previously, it has been reported that curli fimbriae production is crucial for biofilm formation by EHEC strains, particularly ATCC 43895 strain or called EDL933 strain used in this study^4^,^5^,^6^. This qRT-PCR result indicates that clove oil and eugenol strongly reduce the transcription of curli genes, as well as reducing the transcription of several other EHEC genes, including type I fimbriae genes.

### Eugenol reduced fimbriae formation by EHEC

**Figure 5:**
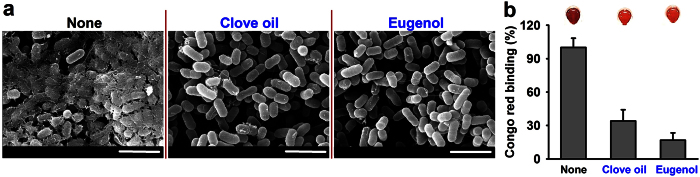
Effects of clove oil and eugenol on curli fimbriae production by EHEC. Fimbriae productions by EHEC cells grown with or without clove oil or eugenol (0.005%) were observed by SEM (**a**). For SEM analysis, cells were cultured in LB broth with or without clove oil or eugenol at 37 °C for 2 h. The scale bar represents 3 μm. Congo red binding assay for the detection of curli fimbriae production (**b**). EHEC cells were grown in LB broth in the presence of Congo red and Commassie brilliant blue at 37 °C for 24 h. Culture supernatants were measured at 490 nm and cell pellets were photographed. Dark red color indicates curli production stained by Congo red and Commassie brilliant blue.

Because fimbriae are important for EHEC biofilm formation^4^,^6^,^27^, and the gene expression of the *csg* operon producing curli fimbriae production was found to be markedly down-regulated by clove oil and eugenol (Fig. 4), we investigated curli fimbriae production by SEM and Congo red that specifically binds curli fimbriae. In-line with our gene expression data, both clove oil and eugenol at 0.005% clearly reduced fimbriae production (Fig. 5a) and specifically curli production (Fig. 5b), which indicates down-regulation of curli genes and of curli fimbriae inhibition underlie the antibiofilm activities of clove oil and of eugenol.

### Biofilm inhibition using a biocompatible polymer coating

**Figure 6:**
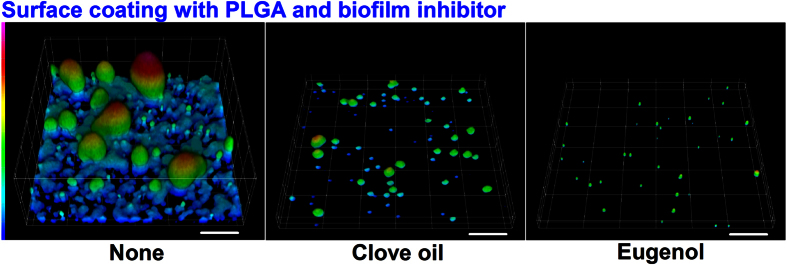
Inhibition of biofilm formation by PLGA coatings containing clove oil or eugenol. Biofilms formed by EHEC/pCM18-GFP were investigated by confocal laser microscopy. Clove oil or eugenol (0.005%, v/v) was incorporated into PLGA and biofilms were grown at 37 °C for 24 h without shaking. Scale bars represent 50 μm. At least three independent experiments were conducted.

To prevent biofilm formation on a solid surface, biofilm inhibitors were incorporated into a biocompatible poly(D,L-lactide-coglycolide) (PLGA; a polymer with many medical, pharmaceutical, and industrial uses). As was expected, PLGA coatings containing clove oil or eugenol (0.005%) markedly inhibited EHEC biofilm formation (Fig. 6). COMSTAT analysis showed that both clove oil and eugenol reduced biofilm biomass, mean thickness, and substratum coverage by ≥90% (Table 3). These results complement observed biofilm reductions observed on polystyrene plates (Figs 1a–c and 2b) and glass (Figs 1d and 2c). Also, the result demonstrates that biocompatible polymer coatings could be potentially used to prevent pathogenic biofilm formation on biomedical and food processing surfaces.

### Clove oil and eugenol reduced EHEC virulence in the nematode model

**Figure 7:**
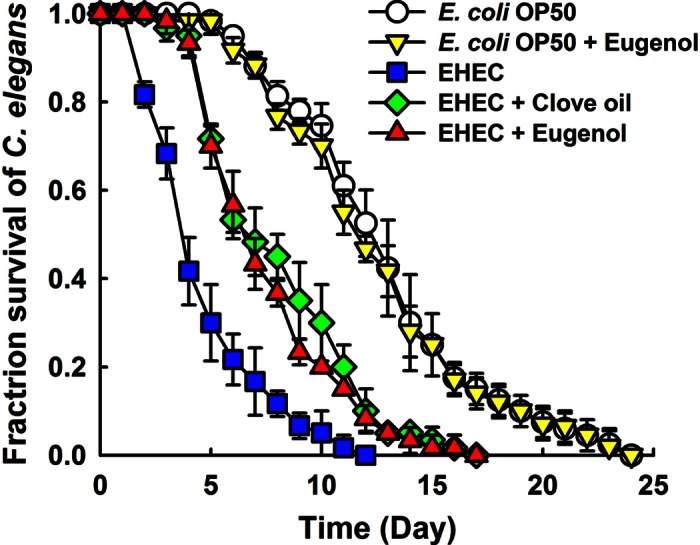
Effects of clove oil or eugenol on the survival of nematodes infected with EHEC. Solid killing assays of *C. elegans* strain *fer-15;fem-1* infected with EHEC or *E. coli* OP50 (control) in the presence of clove oil or eugenol (0.005%, v/v) at 20 °C. The experiment was performed in triplicate (n = 60).

Since EHEC colonizes and replicates in the digestive tract of *C. elegans* and has the ability to kill the nematode^28^, the effects of clove oil and eugenol on EHEC virulence were investigated by assessing *C. elegans* survival. In the agreement with previous results^28^, EHEC significantly reduced the lifespan of *C. elegans* as compared with *E. coli* OP50, which is a common food source for the nematode. Moreover, it was found that clove oil or eugenol at 0.005% prolonged *C. elegans* survival in the presence of EHEC. In fact, the effects of clove oil and eugenol were similar (Fig. 7). This result is in-line with the observed down-regulation of biofilm formation and of the LEE transcriptional regulator *ler* by eugenol (Fig. 4). In addition, to examine the toxicity of eugenol, *C. elegans* survival was investigated using *E. coli* OP50. *E. coli* OP50 fed eugenol at a concentration of 0.005% was found to have no effect on *C. elegans* survival (Fig. 7).

## Discussion

EHEC infection is problematic worldwide because of the lack of an effective therapy and the formation of antibiotic-resistant biofilms by EHEC serotypes. The present study demonstrates that three essential oils (bay, clove, and pimento berry oils) exhibit high antibiofilm activity against EHEC without affecting its planktonic cell growth. Hence, unlike antibiotics that aim to inhibit cell growth, biofilm inhibitors that do not inhibit bacterial growth could reduce the risk of drug resistance. In the present study, identification of the active compound and a chemical structure-activity relationship investigation revealed the antibiofilm activities of eugenol and its derivatives. Furthermore, transcriptional and phenotypic assays provided clues regarding the mechanism of biofilm inhibition and virulence attenuation by eugenol and eugenol-rich oils.

Our investigation of fimbriae production, motility, and laboratory *E. coli* biofilm formation, and qRT-PCR analysis showed that the antibiofilm activities of eugenol and the three oils were due to the suppression of fimbriae production (Figs 3 and 5) and other genes (Fig. 4) in EHEC. It is well known that fimbriae or pili play important role in the biofilm formation of laboratory and pathogenic *E. coli* such as EHEC^9^,^27^,^29^. EHEC strains contain a series of fimbriae such as curli fimbriae (Csg), type I fimbriae (Fim), *E. coli* common pilus (Ecp), F9 fimbriae (Z2200), and other fimbrial proteins^29^. It has been also reported that several phytochemicals, such as, 3-indolylacetonitrile^30^, phloretin^31^, resveratrol (and its dimer viniferin)^32^,^33^, cinnamaldehyde^18^, coumarin^34^, and ginkgolic acids^17^ inhibit EHEC biofilm formation primarily by inhibiting curli fimbriae production. Hence, it appears that fimbriae-reducing ability is not rare in the plant kingdom and that the fimbriae inhibition could be viewed a practical target for suppressing EHEC biofilm formation.

To date, a dozen essential oils have been shown to possess antibacterial and antibiofilm activities against wide range of pathogenic bacteria^13^,^35^,^36^. Previously, eugenol was found to inhibit biofilm formation by *Staphylococcus aureus*^37^, *Candida albicans*^38^, *Listeria monocytogenes*^14^, and EHEC^14^,^18^, and to inhibit quorum sensing of *Pseudomonas aeruginosa*^39^. The present study shows for the first time that eugenol-rich oils (bay, clove, and pimento berry oils) at 0.005% (about 50 μg/ml) have antibiofilm activity against EHEC (Fig. 1), and provides detail of chemical structure-activity relationships and action mechanism involved. Eugenol is used in perfumes, flavorings, and as a local antiseptic and anaesthetic^40^, and is abundant found in clove oil, nutmeg, cinnamon, basil, and bay leaves. This study suggests another application for inexpensive eugenol-rich oils to control EHEC infection.

Our chemical structure-activity relationship study revealed that the C-1 hydroxyl, the C-2 methoxy, and a C-4 alkyl or alkane chain on the aromatic ring of eugenol are required for antibiofilm against EHEC (Fig. 2). Many eugenol derivatives can be synthesized^41^,^42^, and several phytochemicals, such as, zingerones, gingerols, shogaols, and paradols bearing the eugenol motif are found in plant extracts. Thus, further investigations of eugenol derivatives are probably worthwhile to identify more potent antibiofilm and anti-virulence compounds.

EHEC produces potent Shiga-like toxins and encodes the LEE operon genes, which are both required for forming attaching and effacing lesions to host epithelial cells^2^. Furthermore, it has been shown the majority of LEE genes are positively regulated by the *ler* gene (LEE-encoded regulator)^43^. In the present study, eugenol was found to down-regulate the expression of *ler* and *ler*-controlled toxin genes (*espD*, *escJ*, *escR*, and *tir*) and curli-producing genes (*csgABDFG*) (Fig. 4). The pathogenicity of EHEC was attenuated by eugenol and clove oil (Fig. 7), but they did not harm laboratory *E. coli* biofilms (Fig. 3). Therefore, it appears eugenol could be used as a lead compound for the development of biofilm inhibitors and toxin producing inhibitors for fimbriae-rich and LEE-encoding *E. coli* strains, such as, enteropathogenic *Escherichia coli*^44^, uropathogenic *Escherichia coli*^45^, and enteroaggregative *Escherichia coli*^46^.

In the present study, eugenol, eugenol derivatives, and essential oils were found to reduce biofilm formation by and the virulence of EHEC. Furthermore, PLGA coatings containing eugenol or eugenol-rich clove oil effectively prevented EHEC biofilm formation on solid surfaces (Fig. 6). These results suggest that eugenol and its derivatives have potential uses in antivirulence strategies against persistent EHEC infection. In addition, it would be interesting to explore the uses of inexpensive eugenol-rich oils in the food, cosmetics, fishery, agricultural, and environmental industries and in the medical field.

## Materials and Methods

### Bacterial strain, essential oils, and growth conditions

All experiments were conducted at 37 °C in Luria-Bertani (LB) medium, which was used to culture *E. coli* O157:H7 (ATCC 43895, EDL933 strain) and three laboratory *E. coli* K-12 strains (MG1655, BW25113, and TG1). To culture *E. coli* O157:H7/pCM18 tagged with green fluorescent protein, LB broth containing 300 μg/ml of erythromycin was used to maintain the pCM18-GFP plasmid. Bacterial cells were initially streaked from −80 °C glycerol stock on LB plates, grown on plates, and then were cultured from a fresh single colony in LB broth. For phenotypic assays, overnight cultures (stationary phase cells) were inoculated again in LB broth at an initial turbidity of 0.05 at 600 nm. The 83 essential oils (Supplementary Table S1) were obtained from Berjé (Bloomfield, NJ, USA) or Jin Aromatics (Anyang, Gyeonggi Province, Korea). Other chemicals (guaiacol, 4-ethylcatechol, thymol, 2-methoxyhydroquinone, vanillin, carvacrol, methyl eugenol, 2-methoxy-4-propylphenol, isoeugenol, 4-ethylguaiacol, and eugenol) were purchased from Sigma-Aldrich (St. Louis, USA). For cell growth measurements, turbidity was measured at 600 nm using a spectrophotometer (Optizen 2120UV, Mecasys, Korea).

### Crystal-violet static biofilm formation assay

A static biofilm formation assay was performed in 96-well polystyrene plates (SPL Life Sciences, Korea), as previously described^30^. Briefly, overnight cultures were inoculated in LB broth (total volume 300 μl) at an initial turbidity of 0.05 at 600 nm and cultured with or without bay, clove, or pimento berry oils or components for 24 h without shaking at 37 °C. To quantify biofilm formation, cell cultures were washed three times with H_2_O to remove all non-adherent cells. Biofilms were stained with crystal violet for 20 min, rinsed three times with H_2_O, extracted with 95% ethanol, and absorbances were measured at 570 nm. Results are the averages of at least twelve replicate wells.

### Gas chromatograph/mass spectroscopy (GC-MS) analysis

Detailed chemical compositions of the three active essential oils were obtained by GC/MS using an Agilent 6890N GC DB-5 MS fused silica capillary column (30 m × 0.25 m i.d., film thickness 0.25 μm) and a Jeol JMS 700 mass spectrometer. Capillary column details and temperature conditions for the analysis were as previously described^47^. GC-MS was performed using an electron ionization system at 70 eV. Helium was used as the carrier gas at a flow rate of 1 ml/min. The temperatures of the GC injector and MS transfer line were 280 °C and 250 °C, respectively. An initial oven temperature of 50 °C was maintained for 2 min and then increased to 250 °C at a rate of 10 °C/min and this was followed with a holding period at 250 °C for 10 min. Diluted samples (1.0 ml, 1/100 (v/v) in methanol) were injected manually in split-less mode. The relative percentages of oil components were calculated by normalizing peak areas and are expressed as percentages. Components were identified using GC retention times and by matching mass spectra with entries in the Wiley and NIST libraries.

### Confocal laser scanning microscopy and COMSTAT analysis

Bacterial cells (*E. coli* O157:H7/pCM18 tagged with green fluorescent protein) were cultured in glass-bottomed dishes (SPL Life Sciences, Korea) without shaking with or without essential oils or eugenol. Planktonic cells were removed by washing with PBS three times, and biofilms were visualized by excitation using an Ar laser 488 nm (emission wavelengths 500 to 550 nm) under a confocal laser microscope (Nikon eclipse Ti, Tokyo) using a 20 × objective^48^. Color confocal images were constructed using NIS-Elements C version 3.2 (Nikon eclipse). For each experiment, at least 10 random positions in two independent cultures were chosen for microscopic analysis.

To quantify biofilm formation, color confocal images (20 image stacks) were converted to gray scale using ImageJ. COMSTAT biofilm software^49^ was used to determine biomasses (μm^3^ per μm^2^), mean thicknesses (μm), and substratum coverages (%). Thresholding value was fixed for all image stacks, and at least 4 positions and 20 planar images per position were analyzed.

### Fimbriae assay using a Congo red and scanning electron microscopy (SEM)

To measure curli fimbriae production, LB agar medium containing 20 μg/ml Congo red (Sigma), 10 μg/ml Coomassie brilliant blue (Sigma), and 15 g L^–1^ agar was used, as previously described^50^. Curli fimbriae production was visualized after 24 h of incubation at 37 °C on Congo red plates. In addition, a Congo red binding assay was performed, as previously described^51^. Briefly, overnight culture of *E. coli* O157:H7 EDL933 was reinoculated (100:1) with 0.01% eugenol or clove oil in LB medium with 20 μg/ml Congo red and 10 μg/ml Coomassie brilliant blue in 14-ml round bottom tubes and incubated at 37 °C for 24 h with 250 rpm shaking. Incubated cells were collected by centrifugation at 16,600 × g for 15 min. Optical density of the supernatants was measured at 490 nm. Also, SEM was used to observe fimbriae production, as previously described^30^. Briefly, EHEC cells were incubated for 2 h at 37 °C with agitation at 250 rpm, and then re-incubated for 3 h more with or without clove oil or eugenol (0.005%) at 37 °C with shaking. After prompt fixation with glutaraldehyde and formaldehyde, cells were collected by filtering through a 0.45 μm nylon filter under vacuum. The filter was then cut into 0.5 × 0.5 mm squares and post-fixed in sodium phosphate buffer, osmium, ethanol, and isoamyl acetate, and critical-point dried. Specimens were examined using an SEM (S-4100; Hitachi, Japan) at 15 kV and magnifications ranging from 2,000X to 10,000X.

### Swimming and swarming motility

Swimming motility was assayed using 0.3% agar plates containing 1% tryptone and 0.25% NaCl, and swarming motility was assayed using LB broth supplemented with 0.8% glucose and 0.5% agar, as previously described^32^. Essential oils, eugenol or DMSO (the control) were added to motility agar. EHEC was grown to an OD_600_ of 1.0 and then ~0.2 μl aliquots of cultures were placed in motility plates using a sterilized pipette tip. Sizes of swimming halos were measured 16 h later. Each experiment was performed using at least three independent cultures.

### RNA isolation and quantitative real-time RT-PCR

For transcriptional analysis, EHEC was inoculated into 25 ml of LB broth in 250 ml shake flasks at a starting OD_600_ of 0.05, and then cultured at 37 °C for 3 h with agitation (250 rpm) in the presence or absence of clove oil or eugenol (0.005%) for another 2 hours. RNase inhibitor (RNAlater, Ambion, TX, USA) was added to prevent RNA degradation. Total RNA was isolated using a Qiagen RNeasy mini Kit (Valencia, CA, USA).

qRT-PCR was used to investigate the transcription levels of curli genes (*csgA*, *csgB*, *csgD*, *csgF* and *csgG*), type I fimbriae genes (*fimA*, *fimC*, *fimD*, and *fimH*), other fimbriae genes (*ecpA*, *ecpR*, and Z2200), cellulose gene (*bcsA*), motility genes (*flhD*, *fliA*, *motB*, and *qseB*), AI-2 quorum sensing genes (*luxS* and *luxR*), indole-synthesis gene (*tnaA*), shiga-like toxin genes (*stx1* and *stx2*), and LEE-encoded regulator genes (*ler*, *espD*, *escJ*, *escR*, and *tir*) in EHEC treated with or without clove oil or eugenol (0.005%). Gene specific primers were used and *rrsG* was used as a housekeeping control (Supplementary Table S3). The qRT-PCR method used was an adaptation of a previously described method^30^. qRT-PCR was performed using a SYBR Green master mix (Applied Biosystems, Foster City, USA) and an ABI StepOne Real-Time PCR System (Applied Biosystems) on two independent cultures.

### Surface coating with biofilm inhibitors

To fabricate a coating containing biofilm inhibitor, we used biodegradable poly(D,L-lactide-coglycolide) (PLGA) as previously described^52^. Briefly, essential oils or eugenol (final concentration, 0.005% v/v) were mixed into 2% PLGA dissolved in chloroform and 25 μl of these mixtures were applied to slide-glass bottomed dishes to produce a 0.7~0.8 cm diameter coatings, which were then air-dried for 1 h and sterilized by UV exposure for 4 h. To induce biofilm formation on coated glass surfaces, cells (*E. coli* O157:H7/pCM18 tagged with green fluorescent protein; 4 × 10^7 ^CFU/ml) were re-inoculated into LB. Samples were then incubated at 37 °C for 24 h. Planktonic cells were removed by washing with PBS twice, and biofilm cells in PBS buffer were visualized by confocal laser microscopy.

### The *Caenorhabditis elegans* nematode model

The *C. elegans* killing assay was performed as described previously^53^. In brief, *E. coli* O157:H7 was cultured with or without clove oil or eugenol (0.005%) at 37 °C for 18 h, and then 10 μl of *E. coli* O157:H7 was spread onto NGM plates. *E. coli* OP50 was used as a control strain. L4/young adult *fer-15*;*fem-1*^54^ worms (n = 60) were infected by placing them on the lawns. Nematodes were then incubated at 20 °C and scored as alive or dead on a daily basis by gently touching them with a platinum wire. Three independent experiments were conducted.

### Statistical analysis

Sample sizes of all experiments are indicated in ‘Material and Methods’. Results are expressed as means ± standard deviations. The Student’s t-test was used to determine the significances of differences between samples and non-treated controls. Statistical significance was accepted for p values <0.05, and significant changes are indicated by asterisks in figures.

## Additional Information

**How to cite this article**: Kim, Y.-G. *et al.* Essential Oils and Eugenols Inhibit Biofilm Formation and the Virulence of *Escherichia coli* O157:H7. *Sci. Rep.* **6**, 36377; doi: 10.1038/srep36377 (2016).

**Publisher’s note**: Springer Nature remains neutral with regard to jurisdictional claims in published maps and institutional affiliations.

## Supplementary Material

Supplementary Information

## Figures and Tables

**Table 1:** COMSTAT analysis of EHEC biofilms in the presence of essential oils, 4-ethylguaiacol, or eugenol (0.005%).

Chemicals	Volume/Area (μm^3^ μm^−2^)	Mean thickness (μm)	Substratum coverage (%)
None	16.9 ± 2.4	15.1 ± 2.2	89 ± 4
Bay oil	1.9 ± 0.3	2.9 ± 0.9	24 ± 6
Clove oil	1.6 ± 0.5	1.9 ± 0.6	31 ± 8
Pimento berry oil	1.1 ± 0.1	1.4 ± 0.2	21 ± 5
4-Ethylguaiacol	1.0 ± 0.3	1.5 ± 0.4	19 ± 6
Eugenol	0.3 ± 0.1	0.4 ± 0.2	6 ± 2

**Table 2:** GC-MS analysis results for bay, clove, and pimento berry oils.

Compounds^b^	SI^a^			Composition (%)^c^		
	Bay	Clove	Pimento berry	Bay	Clove	Pimento berry
1-Octen-3-ol	800	—	—	1.21	—	—
**Myrcene**	868	—	—	**7**.**93**	—	—
3-Octanol	869	—	—	0.21	—	—
*p*-Cymene	916	—	931	0.51	—	0.37
Limonene	877	—	—	0.89	—	—
Linalool	883	—	—	2.52	—	—
4-Terpineol	904	—	862	0.64	—	0.15
Estragole	891	—	—	0.40	—	—
**Chavicol**	851	—	—	**16**.**48**	—	—
**Eugenol**	901	911	895	**61**.**99**	**77**.**08**	**71**.**10**
**Methyleugenol**	928	—	814	3.40	—	**13**.**67**
δ-Cadinene	854	826	858	0.69	0.36	0.57
Cembrene A	748	—	—	3.12	—	—
*cis*-Caryophyllene	—	851	—	—	0.31	—
*a*-Guaiene	—	773	—	—	0.11	—
***β*****-Caryophyllene**	—	877	899	—	**9**.**10**	**7**.**83**
*β-*Chamigrene	—	785	—	—	0.42	—
Alloaromadendrene	—	772	882	—	0.08	0.24
Eremophilene	—	833	—	—	0.59	—
*a*-Humulene	—	882	880	—	1.73	1.40
*a*-Caryophyllene alcohol	—	535	—	—	0.76	—
(−)-Isolongifolol	—	664	—	—	0.94	—
8,8‘-diapo-20-methoxy carotene-8,8‘-dial	—	867	—	—	0.43	—
Isoeugenyl acetate	—	624	—	—	0.79	—
1,8-Cineole	—	—	848	—		0.29
1-*a*-Terpineol	—	—	837	—		0.09
*β*-Elemene	—	—	868	—		0.29
*γ*-Selinene	—	—	844	—		0.34
*β*-Selinene	—	—	900	—		0.64
*a*-Selinene	—	—	841	—		1.06
(−)-Caryophyllene oxide	—	—	876	—		1.63
Humulene epoxide 2	—	—	721	—		0.15
Juniper camphor	—	—	727	—		0.16

Components present in essential oils at greater than 7% are indicated by bold font.

^a^SI: Library search purity value.

^b^Compounds are listed in order of elution from a DB-5 capillary column.

^c^Percentages were calculated based on normalized FID peak areas.

**Table 3:** COMSTAT analysis of EHEC biofilms on PLGA containing clove oil, or eugenol (0.005%).

Chemicals	Volume/Area (μm^3 ^μm^−2^)	Mean thickness (μm)	Substratum coverage (%)
None	16.2 ± 2.8	16.6 ± 2.3	48 ± 6
Clove oil	0.8 ± 0.2	1.0 ± 0.3	5 ± 1
Eugenol	0.09 ± 0.02	0.09 ± 0.03	0.5 ± 0.1
