# Supplementary material for: Essential Oils and Eugenols Inhibit Biofilm Formation and the Virulence of Escherichia coli O157:H7
Source: Sci Rep. 2016 Nov 3;6:36377. doi: 10.1038/srep36377 (PMC5093407; doi:10.1038/srep36377)
Supplement: Supplementary Information [file srep36377-s1.doc]

**Supplementary Information**

**Essential Oils and Eugenols Inhibit Biofilm Formation and the Virulence of** ***Escherichia coli* O157:H7**

Yong-Guy Kim^1^,*, Jin-Hyung Lee^1^,*, Giyeon Gwon^1^, Soon-Il Kim^2^, Jae Gyu Park^3^, and Jintae Lee^1^**^†^**

^1^School *of Chemical Engineering, Yeungnam University, Gyeongsan 38541, Republic of Korea*

^2^*Nareso Research Center, Seoho-ro 89, Suwon 16614, Republic of Korea*

^3^*Pohang Center for Evaluation of Biomaterials, Pohang Technopark Foundation, Pohang 37668, Republic of Korea*

**Supplementary Figure S1.** Cell growth of EHEC in the presence of essential oils or eugenol at 0.01% (v/v). Planktonic cell growth of EHEC was measured using cell optical densities (OD_600_) in 250-ml flasks agitated at 250 rpm.

**Supplementary Figure S2.** Impacts of bay, clove, or pimento berry oils or eugenol on the swarming (a) and swimming (b) motilities of EHEC. Size (in millimeters) of swarming (c) and swimming (d) motility was determined by measuring the diameter. Swimming motility was measured on 0.3% agar plates containing 1% tryptone and 0.25% NaCl, whereas swarming motility was measured on LB plates containing 0.8% glucose and 0.5% agar after incubation for 24 h. Bay, clove, or pimento berry oils or eugenol (0.005%) were added to motility agar in the beginning of experiment; DMSO (0.1%) was used as the control. Each experiment was performed using three independent cultures.

**
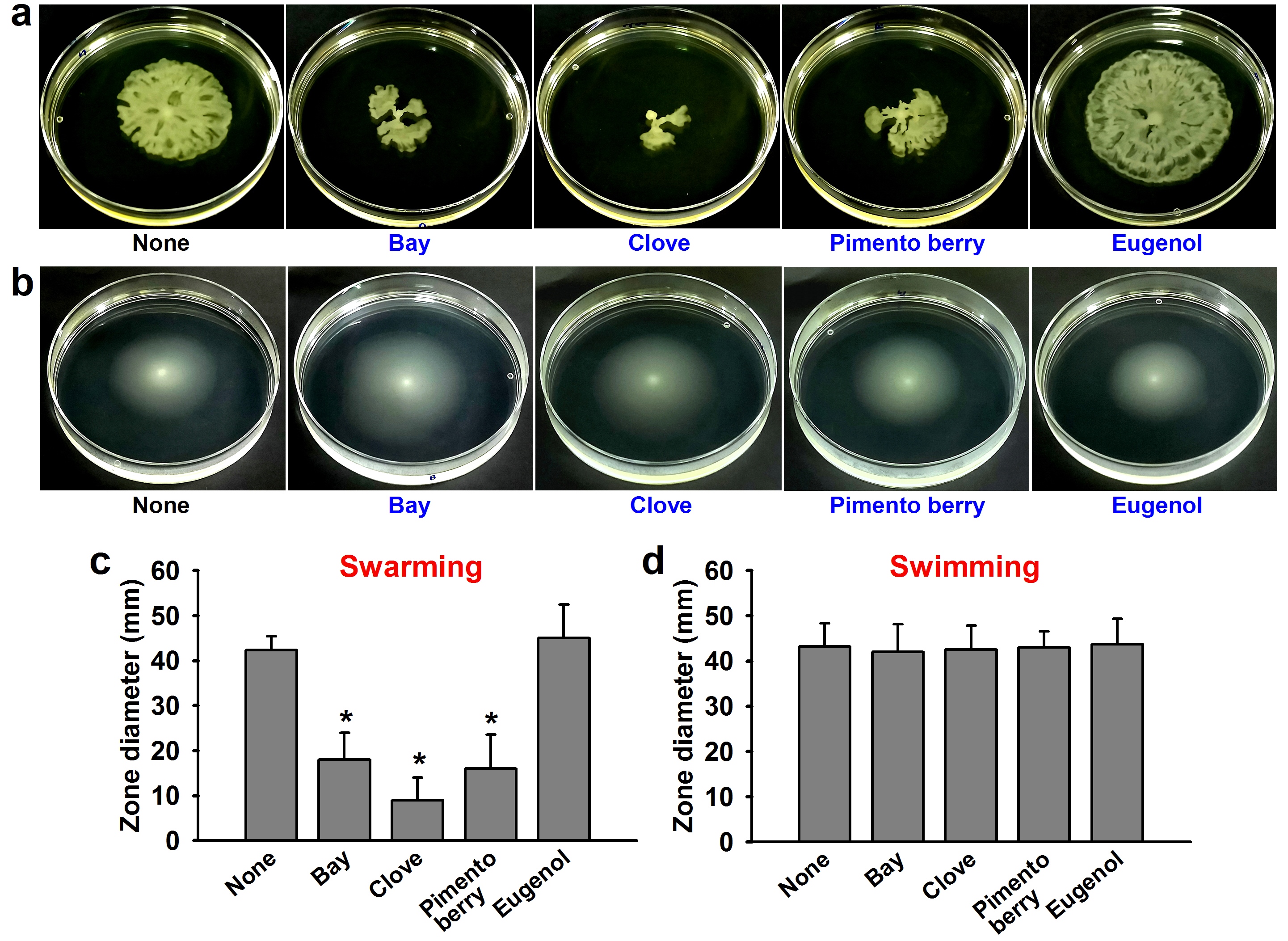
**

**Supplementary Table S1.** Effects of the 83 essential oils on EHEC biofilm formation. Biofilm formation (OD_570_) by EHEC was quantified in the presence of 0.005% of each essential oil after culture for 24 h at 37°C in 96-well plates. Planktonic cell growth was measured at 620 nm in 96-well plates. At least three wells were used, and standard deviations are shown. Percentage ratios represent changes in biofilm formation or growth in the presence of each essential oil versus biofilm formation or growth of the untreated control.

| **Oils** | **Plant species** | **Supplier** | **Biofilm (%)** | **Growth (%)** |
| --- | --- | --- | --- | --- |
| Angelica root | *Angelica archangelica* | Berjé | 80 ± 14 | 91 ± 1 |
| Basil sweet | *Ocimum basilicum* | Berjé | 103 ± 23 | 94 ± 4 |
| **Bay** | ***Pimenta racemosa*** | **Berjé** | **21 ± 9** | **84 ± 1** |
| Bergamot | *Citrus bergamia* | Berjé | 67 ± 24 | 93 ± 5 |
| Black pepper | *Piper nigrum* | Berjé | 86 ± 14 | 96 ± 8 |
| Buchu | *Allium tuberosum* | Berjé | 85 ± 7 | 97 ± 1 |
| Cade | *Juniperus oxycedrus* | Berjé | 67 ± 8 | 97 ± 1 |
| Cananga | *Cananga odorata* | Berjé | 60 ± 7 | 87 ± 5 |
| Caraway seed | *Carum carvi* | Berjé | 86 ± 12 | 96 ± 1 |
| Cardamon | *Elettaria cardamomum* | Berjé | 104 ± 22 | 93 ± 7 |
| Carrot seed | *Daucus carota* | Berjé | 63 ± 7 | 88 ± 5 |
| Cascarilla bark | *Croton eluteria* | Berjé | 93 ± 9 | 97 ± 9 |
| Cedar leaf | *Cedrus libani* | Berjé | 91 ± 8 | 89 ± 1 |
| Cedarwood | [*Calocedrus decurrens*](http://en.wikipedia.org/wiki/Calocedrus_decurrens) | Berjé | 98 ± 10 | 97 ± 7 |
| Celery seed | *Apium graveolens* | Berjé | 90 ± 10 | 93 ± 6 |
| Chamomile | [*Matricaria chamomilla*](http://en.wikipedia.org/wiki/Matricaria_chamomilla) | Berjé | 83 ± 5 | 95 ± 2 |
| Chamomile Roman | [*Chamaemelum nobile*](http://en.wikipedia.org/wiki/Chamaemelum_nobile) | Berjé | 83 ± 18 | 94 ± 1 |
| Cinnamon bark | [*Cinnamomum cassia*](http://en.wikipedia.org/wiki/Cinnamomum_verum) | Berjé | 16 ± 6 | 86 ± 2 |
| Citronella | *Cymbopogon nardus* | Berjé | 96 ± 3 | 88 ± 1 |
| Citronella java | *Cymbopogon winterianus* | Berjé | 82 ± 7 | 92 ± 2 |
| Clary sage | *Salvia sclarea* | Berjé | 87 ± 13 | 99 ± 4 |
| **Clove** | ***Syzygium aromaticum*** | **Berjé** | **22 ± 18** | **77 ± 1** |
| Coriander herb | *Coriandrum sativum* | Berjé | 94 ± 18 | 97 ± 5 |
| Cypress | *Cupressus sempervirens* | Berjé | 80 ± 13 | 94 ± 3 |
| Davana | *Artemisia pallens* | Berjé | 85 ± 10 | 96 ± 1 |
| Dillseed | *Anethum graveolens* | Berjé | 114 ± 13 | 98 ± 5 |
| Eucalyptus | [*Eucalyptus globulus*](http://en.wikipedia.org/wiki/Eucalyptus_globulus) | Berjé | 106 ± 15 | 95 ± 5 |
| Eucalyptus | *Eucalyptus polybractea* | Berjé | 92 ± 17 | 93 ± 6 |
| Fennel seed | *Foeniculum vulgare* | Berjé | 85 ± 6 | 94 ± 1 |
| Frankincense | *Boswellia carterii* | Berjé | 69 ± 9 | 87 ± 8 |
| Galbanum | *Ferula gummosa* | Berjé | 85 ± 8 | 91 ± 6 |
| Garlic | *Allium sativum* | Berjé | 101 ± 8 | 99 ± 3 |
| Geranium | *Pelargonium odorantissimum* | Berjé | 86 ± 12 | 88 ± 7 |
| Ginger | *Zingiber officinale* | Berjé | 78 ± 15 | 106 ± 5 |
| Helichrysum | *Helichrysum italicum* | Berjé | 110 ± 4 | 99 ± 10 |
| Horseradish | *Armoracia rusticana* | Berjé | 102 ± 11 | 99 ± 5 |
| Hyssop | *Hyssopus officinalis* | Berjé | 131 ± 11 | 99 ± 11 |
| Juniperberry | *Juniperus communis* | Berjé | 66 ± 10 | 84 ± 6 |
| Lavender | [*Lavandula angustifolia*](http://en.wikipedia.org/wiki/Lavandula_angustifolia) | Berjé | 110 ± 10 | 102 ± 1 |
| Lemon | *Citrus limon* | Berjé | 120 ± 1 | 94 ± 1 |
| Lemon eucalyptus | *Eucalyptus citriodora* | Berjé | 89 ± 5 | 101 ± 4 |
| Lemongrass | *Cymbopogon citratus* | Berjé | 52 ± 3 | 84 ± 2 |
| Lime | *Citrus glauca* | Berjé | 73 ± 12 | 86 ± 1 |
| Litsea | *Litsea cubeba* | Berjé | 81 ± 1 | 98 ± 1 |
| Lovage root | *Levisticum officinale* | Berjé | 57 ± 9 | 95 ± 1 |
| Mace | *Myristica fragrana* | Berjé | 115 ± 6 | 98 ± 7 |
| Majoram | [*Origanum majorana*](http://en.wikipedia.org/wiki/Origanum) | Berjé | 67 ± 7 | 90 ± 3 |
| Mandarin | *Citrus reticulate* | Berjé | 77 ± 13 | 92 ± 1 |
| Melissa | *Melissa officinalis* | Berjé | 71 ± 5 | 89 ± 1 |
| Mustard | [*Brassica juncea*](http://en.wikipedia.org/wiki/Brassica_juncea) | Berjé | 57 ± 11 | 91 ± 9 |
| Myrrh | *Commiphora myrrha* | Berjé | 69 ± 6 | 95 ± 8 |
| Myrtle | [*Myrtus communis*](http://en.wikipedia.org/wiki/Myrtus_communis) | Berjé | 87 ± 16 | 94 ± 7 |
| Neroli | *Citrus aurantium* | Berjé | 72 ± 10 | 89 ± 6 |
| Niaouli | *Melaleuca viridiflora* | Jin-A | 89 ± 8 | 91 ± 1 |
| Nutmeg | *Myristica fragrans* | Jin-A | 65 ± 9 | 89 ± 3 |
| Oregano | *Origanum vulgare* | Jin-A | 42 ± 12 | 76 ± 1 |
| Origanum | *Origanum vulgare* | Jin-A | 81 ± 23 | 79 ± 1 |
| Palmarosa | *Cymbopogon martinii* | Jin-A | 56 ± 19 | 93 ± 1 |
| Parsley seed | *Petroselinum crispum* | Jin-A | 69 ± 5 | 89 ± 5 |
| Patchouli | *Pogostemon patchouli* | Jin-A | 101 ± 15 | 97 ± 1 |
| Pennyroyal | [*Mentha pulegium*](http://en.wikipedia.org/wiki/Mentha_pulegium) | Jin-A | 66 ± 11 | 97 ± 1 |
| Peppermint | *Menthe x piperita* | Berjé | 70 ± 15 | 96 ± 4 |
| Petitgrain | *Citrus aurantium* | Berjé | 86 ± 13 | 95 ± 5 |
| **Pimento berry** | ***Pimenta officinalis*** | **Berjé** | **10 ± 4** | **78 ± 1** |
| Pine needle | [*Pinus sylvestris.*](http://en.wikipedia.org/wiki/Pinus_sylvestris) | Berjé | 81 ± 5 | 92 ± 1 |
| Rosemary | *Rosmarinus officinalis* | Berjé | 72 ± 1 | 91 ± 6 |
| Rosewood | *Aniba rosaeodora* | Berjé | 66 ± 6 | 89 ± 5 |
| Sage | *Salvia officinalis* | Berjé | 108 ± 1 | 96 ± 8 |
| Sage Spanish | *Salvia fruticosa* | Berjé | 73 ± 11 | 98 ± 1 |
| Sandalwood | *Santalum album* | Berjé | 78 ± 12 | 84 ± 8 |
| Sassafras | [*Sassafras albidum,*](http://en.wikipedia.org/wiki/Sassafras_albidum) | Berjé | 87 ± 9 | 94 ± 1 |
| Savory | *Satureja hortensis* | Berjé | 90 ± 12 | 85 ± 1 |
| Star anise | [*Illicium verum*](http://en.wikipedia.org/wiki/Illicium_verum) | Berjé | 75 ± 9 | 94 ± 7 |
| Tagette | *Tagetes glandulifera* | Berjé | 62 ± 4 | 90 ± 5 |
| Tangerine | *Citrus tangerina* | Berjé | 85 ± 12 | 105 ± 5 |
| Tarragon | *Artemisia dracunculus* | Berjé | 62 ± 4 | 91 ± 5 |
| Tea tree | *Melaleuca alternifolia* | Berjé | 112 ± 15 | 95 ± 5 |
| Thyme red | *Thymus vulgaris* | Berjé | 85 ± 8 | 91 ± 3 |
| Valerian | *Valeriana officinalis* | Sigma | 116 ± 10 | 99 ± 1 |
| Vetiver Haiti | [*Cymbopogon martinii*](http://en.wikipedia.org/wiki/Cymbopogon_martinii) | Berjé | 74 ± 11 | 90 ± 3 |
| Wintergreen | [*Gaultheria procumbens*](http://en.wikipedia.org/wiki/Gaultheria_procumbens) | Berjé | 94 ± 9 | 98 ± 6 |
| Wormwood | [*Artemisia absinthium*](http://en.wikipedia.org/wiki/Artemisia_absinthium) | Berjé | 83 ± 18 | 101 ± 10 |
| Yarrow | *Achillea millefolium* | Berjé | 72 ± 7 | 91 ± 9 |

**Supplementary Table S2.** Transcriptional change of fimbrial genes in EHEC cells with clove oil or eugenol.

| **Fimbriae**  **& cellulose** | **Genes** | **Description** | **Fold change**  **in qRT-PCR**  **Clove oil Eugenol** | |
| --- | --- | --- | --- | --- |
| **Curli fimbriae** | *csgA* | Curli subunit, major curli subunit | -86 ± 15 | -49 ± 35 |
|  | *csgB* | Curli nucleator protein curlin CsgB | -155 ± 83 | -33 ± 27 |
|  | *csgD* | Transcriptional activator for *csgBA* | -7 ± 2 | -8 ± 4 |
|  | *csgF* | Protein required for curli nucleation by CsgB | -13 ± 7 | -13 ± 3 |
|  | *csgG* | Pore-forming lipoprotein for curli subunit secretion | -9 ± 3 | -8 ± 4 |
| **Type I**  **fimbriae** | *fimA* | Fimbrin type 1, major structural subunit | -2.1 ± 0.2 | +1.2 ± 1.0 |
|  | *fimC* | Periplasmic chaperone for type 1 fimbriae | -1.0 ± 0.3 | -1.8 ± 0.4 |
|  | *fimD* | Assembly and anchoring of type 1 fimbriae | -1.4 ± 0.3 | -2.5 ± 0.3 |
|  | *fimH* | Minor type 1 fimbrial subunit | -3.6 ± 1.0 | -2.6 ± 1.3 |
| **Other fimbriae** | *ecpA* | *E. coli* common pilus (Ecp) | -1.3 ± 0.1 | -1.3 ± 0.3 |
|  | *ecpR* | Transcriptional regulator for the *ecp* operon | -3.0 ± 1.1 | -2.5 ± 0.6 |
|  | *Z2200* | F9 fimbrial subunit | +1.1 ± 0.3 | -1.5 ± 0.1 |
| **Cellulose** | *bcsA* | Celullose synthase | -1.4 ± 0.8 | -3.0 ± 0.1 |

**Supplementary Table S3.** Sequences of the primers used for quantitative RT-PCR.

| **Gene** | **Primer** |
| --- | --- |
| *rrsG* | Forward 5'-TAT TGC ACA ATG GGC GCA AG-3' |
|  | Reverse 5'-ACT TAA CAA ACC GCC TGC GT-3' |
| *csgA* | Forward 5'-AGA TGT TGG TCA GGG CTC AG-3' |
|  | Reverse 5'-CGT TGT TAC CAA AGC CAA CC-3' |
| *csgB* | Forward 5’-ATC AGG CAG CCA TAA TTG GT-3’ |
|  | Reverse 5’-CCA TAA GCA CCT TGC GAA AT-3’ |
| *csgD* | Forward 5’-CCG CTT GTG TCC GGT TTT-3’ |
|  | Reverse 5’-GAG ATC GCT CGT TCG TTG TTC-3’ |
| *csgF* | Forward 5’-TTG AAA CAC CCT CAG CGT TA-3’ |
|  | Reverse 5’-CTG GAT GGT CGA GGT TTG TC-3’ |
| *csgG* | Forward 5'-CCG GGT AGG GTT TAA ATT TG-3' |
|  | Reverse 5'-GCA GCG CTT ATT TCT TTT GG-3' |
| *fimA* | Forward 5'-CCA GTT CTG CTG TCG GTT TT-3' |
|  | Reverse 5'-TCA GGG TTG TTT GCT CAC TG-3' |
| *fimC* | Forward 5'-AGG GCA AAA ACA AGT GCA AC-3' |
|  | Reverse 5'-TAA ACT TTC CCG GTC CTG TG-3' |
| *fimD* | Forward 5'-TAT ATG GCA ACG CGT GAT GT-3' |
|  | Reverse 5'-CGC TGC TGA CCA ACA TCT AA-3' |
| *fimH* | Forward 5'-CGT GCT TAT TTT GCG ACA GA-3' |
|  | Reverse 5'-AGG AAT TGG CAC TGA ACC AG-3' |
| *ecpA* | Forward 5'-CGC GGA TCC ATG AAA AAA AAG GTT CTG GC-3' |
|  | Reverse 5'-CGC GAA TTC TAA CTG GTC CAG GTC GCG TCG-3' |
| *ecpR* | Forward 5'-ACA TCT GGT CTC CCC ATG AC-3' |
|  | Reverse 5'-TTT ACC GCG GAT AAC CAT TC-3' |
| *Z2200* | Forward 5'-TTG CGA TAC CAA TGT TGC AT-3' |
|  | Reverse 5'-TGG AAC GGA ATG GTA TTG GT-3' |
| *bcsA* | Forward 5'-TGC TGC TTC ATG CCT ACA TC-3' |
|  | Reverse 5'-CTT TGG CGG TGA CGT TAA AT-3' |
| *flhD* | Forward 5'-TGC ATA CCT CCG AGT TGC TG-3' |
|  | Reverse 5'-GCG TGT TGA GAG CAT GAT GC-3' |
| *fliA* | Forward 5'-TTA GGG ATC GAT ATT GCC GAT T-3' |
|  | Reverse 5'-CGT AGG AGA AGA GCT GGC TGT T-3' |
| *motB* | Forward 5'-CAG GGG GAA GTG AAT AAG CA-3' |
|  | Reverse 5'-TTC TAA ACA TCG GGC GAT TC-3' |
| *qseB* | Forward 5'-GGC GAA CCC TTA ACA CTG AA-3' |
|  | Reverse 5'-CCA TGC ACG GTA CGA ATA AA-3' |
| *luxS* | Forward 5’-CAT ACC CTG GAG CAC CTG TT-3’ |
|  | Reverse 5’-TGA TCC TGC ACT TTC AGC AC-3’ |
| *luxR* | Forward 5'-GGC CGA ACT CGT AAA ATG G-3' |
|  | Reverse 5'-ATT AAG TTT CGC CGG GTG AC-3' |
| *tnaA* | Forward 5'-TGA AGA AGT TGG TCC GAA TAA CGT G-3' |
|  | Reverse 5'-CTT TGT ATT CTG CTT CAC GCT GCT T-3' |
| *stx1* | Forward 5'-GTC ACA GTA ACA AAC CGT AAC A-3' |
|  | Reverse 5'-TCG TTG ACT ACT TCT TAT CTG GA-3' |
| *stx2* | Forward 5'-GTT CCG GAA TGC AAA TCA GT-3' |
|  | Reverse 5'-CGG CGT CAT CGT ATA CAC AG-3' |
| *ler* | Forward 5’-CGA CCA GGT CTG CCC TTC T-3’ |
|  | Reverse 5’-GCG CGG AAC TCA TCG AAA-3’ |
| *espD* | Forward 5’-AAT TGT TGG CCA GGT CTT TG-3’ |
|  | Reverse 5’-GCT TGC GGC CAT AGA TAT TC-3’ |
| *escJ* | Forward 5’-CCA ATG ATG TCA ATG TTT CCA AA-3’ |
|  | Reverse 5’-GCG CGA ACA AAA TCC TCT TT-3’ |
| *escR* | Forward 5’-GCC AGC CTC CAA CAA GAA TG-3’ |
|  | Reverse 5’-ATT GGC CTT GGG TAT GAT GAT G-3’ |
| *tir* | Forward 5’-ACT TCC AGC CTT CGT TCA GA-3’ |
|  | Reverse 5’-TTC TGG AAC GCT TCT TTC GT-3’ |
